# Convergent gut microbiome adaptation and pervasive antibiotic resistome in Qinghai–Tibet Plateau passerines

**DOI:** 10.3389/fmicb.2025.1733974

**Published:** 2026-02-04

**Authors:** Shunan Shi, Jiancheng Qi, Wenxuan Peng, Xiaodong Su, Peng Chen, Shoubiao Xu, Sheng Li, Long Ma, Wenlong Wang, Ke Jiang, Zhiguo Liu, Wei Li, Haoming Xiong, Yongshun Wang

**Affiliations:** 1 Department of Public Health, Qinghai University Medical College, Xining, China; 2 Plague Prevention and Control Section of the Qinghai Provincial Institute for Endemic Disease Prevention and Control, Xining, China; 3 Specialized Laboratory of Yersinia Pestis, Qinghai Institute for Endemic Disease Prevention and Control, Xining, China; 4 Tianjun County Center for Disease Control and Prevention, Haixi, China; 5 National Institute for Communicable Disease Control and Prevention, Chinese Center for Disease Control and Prevention, Beijing, China

**Keywords:** Antibiotic resistance genes (ARGs), gut microbiome, metagenomic, nonmigratory passerines, Qinghai–Tibet Plateau

## Abstract

**Introduction:**

The Qinghai-Tibet Plateau, an extreme high-altitude ecosystem, presents a unique model for studying host-microbe-environment coevolution under environmental stress. However, the role of resident wildlife, particularly non-migratory passerines, as reservoirs and vectors for cross-boundary antibiotic resistance gene (ARG) dissemination remains poorly understood.

**Methods:**

Here, through metagenomic analysis of two endemic passerines (Pseudopodoces humilis and Pyrgilauda ruficollis) and their habitats.

**Results:**

We reveal convergent adaptations in their gut microbiomes, dominated by *Actinomycetota*, *Pseudomonadota* and *Bacillota*. Functional enrichment in carbohydrate metabolism and genetic information processing underpins host energy optimization in extreme high-altitude environments. Critically, these birds constitute a major reservoir of ARGs, harboring 153 antibiotic resistance ontologies (AROs) with nearly universal resistance to clinical antibiotic classes. The core resistome—comprising glycopeptide (*van* clusters), fluoroquinolone, and tetracycline resistance genes—reflects anthropogenic contamination amplified by environmental persistence. Environmental transmission pathways were unequivocally demonstrated via 47 AROs shared between avian hosts and proximal matrices (soil/grass), coupled with livestock-derived antibiotic influx through excreta, establishing the plateau as a hotspot for resistance gene flux. Strikingly, “low-abundance–high-resistance” taxa (*Pseudomonadota*, *Actinomycetota*, and *Bacillota*; ≤30% abundance but >80% ARG contribution) drive resistome plasticity, potentially facilitated by horizontal gene transfer.

**Discussion:**

Our findings redefine resident passerines as sentinels of ecosystem health and bridges for cross-boundary antimicrobial resistance (AMR) spread. Mitigating global AMR thus necessitates interdisciplinary strategies targeting environmental reservoirs (e.g., regulating livestock antibiotic use) and monitoring avian-mediated gene flow.

## 1 Introduction

The Qinghai–Tibet Plateau, the highest-altitude ecosystem on Earth, is characterized by a unique combination of hypobaric hypoxia, low temperatures, and intense ultraviolet radiation, which collectively drive coevolution between native species and their symbiotic microbial communities (Liu et al., 2016; Li et al., 2018). As a critical symbiotic interface, the gut microbiota facilitates host adaptation to extreme environments through two regulatory mechanisms: metabolic optimization and immune homeostasis maintenance (Tang et al., 2024). The assembly of microbial communities results from the tripartite interplay of host genetics, dietary substrates, and environmental selection pressures (Fragiadakis et al., 2019; Orkin et al., 2019; Kešnerová et al., 2020). Emerging evidence indicates that indigenous plateau mammals (e.g., Yaks, plateau pikas and Tibetan antelope) exhibit seasonally stratified remodeling of the gut microbiota, enabling ecological niche-specific energy harvesting strategies (cellulose degradation in winter vs. protein catabolism in summer, Ma et al., 2019; Fan et al., 2022). These findings elucidate the microbe-mediated mechanisms underlying host extremophilic adaptation.

Meanwhile, tens of thousands of tons of antibiotics are consumed annually in healthcare, livestock, and agricultural settings worldwide (Van Boeckel et al., 2015). This anthropogenic pressure has accelerated the global dissemination of antibiotic-resistant bacteria (ARB) and ARGs, constituting a planetary-scale threat to ecological integrity and One Health security (Knapp et al., 2010). Critically, the increasing prevalence of multidrug-resistant (MDR) pathogens has established AMR-associated infections as a leading cause of mortality worldwide, including AIDS-related deaths (Sommer et al., 2009) Environmental compartments—including soil matrices, aquatic systems, and animal waste reservoirs—serve as ARG hotspots, enabling cross-ecosystem transmission through horizontal gene transfer (HGT), hydrological connectivity, and mobile vectors (e.g., migratory avifauna) (Allen et al., 2010; Smillie et al., 2011). Mounting experimental evidence has demonstrated that gut microbiomes function as critical reservoirs for persistent ARB populations and transmissible ARG repertoires (Knapp et al., 2010; Hu et al., 2013). Notably, wild birds represent underappreciated mobile vectors facilitating the intercontinental spread of evolving ARB lineages and novel ARG combinations through latitudinal migration networks (Allen et al., 2010).

Compared with those of mammals, avian gut microbiomes exhibit lower stability and greater plasticity (Hird, 2017), traits strongly influenced by selective pressures from their complex life-history strategies, including dietary flexibility, flight-associated physiological adaptations, and long-distance migrations (García-Amado et al., 2018). Metagenomic analyses by Lin et al. (2020) revealed that migratory birds harbor mobile genetic elements (MGEs) and demonstrate high coselection potential for ARG, increasing the risk of environmental contamination. Notably, endemic Tibetan Plateau species such as *Pseudopodoces humilis* and *Pyrgilauda ruficollis* have evolved hypoxia-responsive mechanisms to thrive in extremely high-altitude environments (3200–4,550 m) (Yang et al., 2006). Despite these adaptations, current avian microbiome research remains disproportionately focused on low-altitude and migratory waterfowl. Field sampling challenges have hindered investigations of plateau-dwelling resident birds, which serve dual roles as AMR reservoirs and bioindicators of ecosystem health in low-disturbance regions. The distribution patterns of ARGs and host–microbe coadaptation mechanisms in these birds’ unique habitats remain poorly characterized, underscoring the urgent need for targeted studies to address the increasing global threat of antibiotic resistance.

Previous studies have characterized the gut microbiota of avian species such as *Charadrius altrifrons*, *Charadrius alexandrinus*, and *Passer montanus* using 16S rRNA gene amplicon sequencing and culture-dependent methods (Sun et al., 2023, 2024). However, these approaches offer limited resolution of microbial community dynamics and fail to comprehensively resolve ARG profiles in wild birds. Recent advances in high-throughput metagenomics have enabled systematic annotation of functional pathways and ARG identification across host-associated and environmental microbiomes (Kohl, 2012; Turaev and Rattei, 2016). To address these gaps, we selected two plateau-endemic resident birds, the Ground Tit (*Pseudopodoces humilis*) and the Rufous-necked Snowfinch (*Pyrgilauda ruficollis*), as model systems. Leveraging metagenomic sequencing, we aimed to (1) elucidate functional regulatory networks governing host–microbe interactions under extreme environmental stress, (2) characterize community-wide ARG distribution patterns and horizontal transmission potential, and (3) evaluate the role of resident birds as ARG reservoirs and the implications for ecosystem health. Our findings advance the understanding of microbial evolutionary adaptation in extremely high-altitude ecosystems while providing actionable insights into the wildlife-mediated global dissemination of antimicrobial resistance, thereby informing strategies to mitigate this pressing public health challenge.

## 2 Materials and methods

### 2.1 Sampling

Sampling efforts were carried out at three sites in Tianjun County, Menyuan County and Xinghai County, Qinghai Province, from July to August 2024 (Supplementary Figure S1; Supplementary Table S1). We trapped *Pseudopodoces humilis* (in text abbreviation: PH group) and *Pyrgilauda ruficollis* (in text abbreviation: PR group) using live trapping and locked them in sterilized cages (typically for less than 30 min) until defecation occurred, after which they were immediately released at the capture site. Fresh feces were collected in 2-mL tubes (Kejin, China) and immediately frozen in liquid nitrogen before being sent for analysis to the Qinghai Provincial Institute for Endemic Disease Prevention and Control, Xining, China. The fecal samples were collected at a minimum distance interval of five meters to ensure that all the fresh droppings were expelled from different individuals, and the captured individuals were marked with nontoxic avian leg bands prior to release. Composite soil samples were collected from a 10 × 10 m area around each bird capture site using a sterilized shovel by pooling five random soil cores (20 cm depth, 5 cm diameter), while the above-ground parts of dominant grass species were sampled from the same area using sterile scissors and forceps. All samples were immediately placed in sterile 50-mL tubes, flash-frozen in liquid nitrogen, and stored at −80 °C for subsequent DNA analysis. In total, we collected 16 PH and 16 PR fecal samples, 15 soil samples and 6 grass samples.

### 2.2 DNA extraction and sequencing

Metagenomic DNA was isolated from approximately 0.25 g samples using a TIANamp Soil DNA Kit (Tiangen Biotech; Beijing; China) following the manufacturer’s instructions. The DNA concentration and quality were assessed with an Agilent 5,400 instrument. The high-quality DNA was randomly fragmented into segments of approximately 350 bp using a Covaris ultrasonic disruptor to construct the library. Using the Rapid Plus DNA Lib Prep Kit for Illumina (RK20208), sequencing libraries were constructed through end repair, addition of A-tails, ligation of sequencing adapters, purification, and PCR amplification. All libraries were then subjected to 2 × 150 bp paired-end sequencing on the BGISEQ DNBSEQ-T7 platform (Novogene, Beijing, China).

### 2.3 Sequence analyses and metagenome assembly

The raw BGISEQ paired-end sequencing data were preprocessed using Fastp (v0.23.1). The preprocessing pipeline consisted of adapter trimming, followed by quality filtering. During the filtering step, read pairs were discarded if either read contained (a) residual adapter sequences; (b) > 10% ambiguous bases (N); or (c) > 50% low-quality bases (Phred score <5). Considering the possibility of host contamination in samples, clean data were subjected to BLAST analysis against the host database to filter out reads of host origin. Bowtie2 software (v2.5.4) was used with the following default parameter settings: --end-to-end, --sensitive, -I 200, and -X 400 (Karlsson et al., 2012, 2013; Scher et al., 2013). MEGAHIT software (v1.2.9) was used for assembly analysis of the clean data, with the following assembly parameter settings: --presets meta-large (--end-to-end, --sensitive, -I 200, -X 400) (Karlsson et al., 2013; Nielsen et al., 2014), and scaftigs without N were obtained by breaking the resulting scaffolds from the N junction (Qin et al., 2010; Li et al., 2015).

### 2.4 Gene prediction and construction of the nonredundant gene set

With the default parameters, MetaGeneMark (v2.1) was used to perform ORF prediction for scaftigs (≥ 500 bp) of each sample (Karlsson et al., 2012; Li et al., 2014; Mende et al., 2012; Oh et al., 2014; Qin et al., 2014), and sequences with a length of less than 100 nt in the prediction results were filtered out (Qin et al., 2010; Zhu et al., 2010; Nielsen et al., 2014; Zeller et al., 2014; Sunagawa et al., 2015). For the ORF prediction results, CD-HIT software (v4.5.8) was used to eliminate redundancy (Li and Godzik, 2006; Fu et al., 2012) and obtain the nonredundant initial gene catalogue (representing the nucleotide sequences of successive, non-redundant genes) (Zeller et al., 2014), with parameter settings: -c 0.95, -G 0, -aS 0.9, -g 1, -d 0 (Li et al., 2014; Qin et al., 2014). Clean data from each sample were aligned to the initial gene catalogue by using Bowtie2 to calculate the number of reads of the genes in each sample alignment, with the following parameter settings: --end-to-end, --sensitive, -I 200, -x 400 (Qin et al., 2010; Li et al., 2014). Genes with <= 2 reads in each sample were filtered out to finally determine the gene catalogue (unigenes) for subsequent analysis (Zeller et al., 2014). On the basis of the abundance of each gene in the gene catalogue in each sample, the abundance information of each unigene in each sample was statistically analyzed. To ensure accurate characterization of the prokaryotic microbiome, all sequences taxonomically assigned to eukaryota and viruses were excluded from downstream analyses, including taxonomic profiling, functional annotation, and antibiotic resistance gene screening.

### 2.5 Gene taxonomic prediction

DIAMOND software (v2.1.9) (Buchfink et al., 2015) was used for alignment of unigene sequences with the Micro_NR database, which included sequences from bacteria, fungi, archaea, and viruses extracted from the NCBI NR database (https://www.ncbi.nlm.nih.gov/). The alignment was performed using the BLASTP algorithm with a parameter setting of 1e-5 (Karlsson et al., 2013). From the alignment results of each sequence, the one with evalue <= min. Evalue *10 was selected. Since each sequence may have multiple alignment results, the lowest common ancestor (LCA) algorithm [applied to the systematic taxonomy of MEGAN software (https://en.wikipedia.org/wiki/Lowest_common_ancestor)] was adopted to determine the species annotation information of the sequence (Huson et al., 2011). In addition to the results of the lowest common ancestor (LCA) annotation and gene abundance table, the abundance of each sample at each taxonomic level and the corresponding gene abundance tables were acquired. The abundance of a species in a sample was equal to the sum of the abundance of those genes annotated as that species. Microbiome analyses were conducted using a standardized bioinformatics pipeline. Taxonomic abundance profiles across hierarchical levels were visualized through relative abundance profiles and Bray–Curtis distance-based clustering trees implemented in Perl SVG (v5.18.2).

### 2.6 Functional gene annotation

Functional annotation of the unigenes was performed using DIAMOND (v2.1.9) with BLASTP alignment against the Kyoto Encyclopedia of Genes and Genomes (KEGG) (v2023.1) (Kanehisa et al., 2006, 2017) and Pathogen–Host Interaction (PHI) database (v4.12) by applying an e-value cut-off of 1e−5 (Li et al., 2014; Feng et al., 2015). From the alignment results of each sequence, the best BLAST hit results were selected for subsequent analysis (Qin et al., 2012, 2014; Li et al., 2014; Bäckhed et al., 2015). The relative abundance at different functional levels was calculated according to the alignment results (the relative abundance at each functional level was equal to the sum of the relative abundance of genes annotated at that functional level) (Karlsson et al., 2012; Li et al., 2014). For antibiotic resistance profiling, the unigenes were aligned to the Comprehensive Antibiotic Resistance Database (CARD) (https://card.mcmaster.ca/) (Martínez et al., 2015) using Resistance Gene Identifier (RGI) software (v6.0.2) (Jia et al., 2017) provided by the CARD database (RGI built-in BLASTP, default evalue < 1e-30) (McArthur et al., 2013). The relative abundance of each ARO was calculated according to the RGI alignment result and unigene abundance information. To reveal the relationship between microbial composition and the resistome, Circos (v0.64) generated visualization of abundance distribution circle map. Resistance genes (unigenes annotated as ARO) and species attribution analysis of the resistance mechanism were carried out (some AROs with long names are abbreviated as the first three words plus underlines).

### 2.7 Statistical analysis

Venn diagrams were generated using the R package VennDiagram (v3.0.3). Alpha diversity, including the Chao1 richness, observed_species and Shannon indices, were computed using the R package vegan (v2.15.3). Differences in alpha diversity between groups were assessed using the non-parametric Kruskal-Wallis test. Beta diversity was evaluated based on Bray–Curtis dissimilarity matrices, and group differences were tested using permutational multivariate analysis of variance (PERMANOVA) with 9,999 permutations via the adonis2 function in the vegan package. Principal coordinate analysis (PCoA) was performed to visualize beta diversity patterns using the R packages extrafont, ggplot2, and grid (v2.15.3). LEfSe software (v1.0) was used for Linear Discriminant Analysis Effect Size (LEfSe) analysis (LDA Score was 4 by default) (Segata et al., 2011). Statistical significance was set at *p* < 0.05.

## 3 Results

### 3.1 Bacterial community profile

In total, 53 metagenomes were sequenced using the BGISEQ DNBSEQ-T7 platform (2 × 150 bp). High-throughput sequencing of the microbial DNA samples generated 391.4 Gb of high-quality clean data from the 53 samples. On average, 7.38 Gb of data were obtained per sample (Supplementary File 1). Furthermore, after *de novo* assembly, 1306.62 Mb of scaftigs (with total lengths ranging from 0.94 to 52.13 Mb) were generated, with an N50 of 76,426 bp (Supplementary File 2).

**Figure 1:**
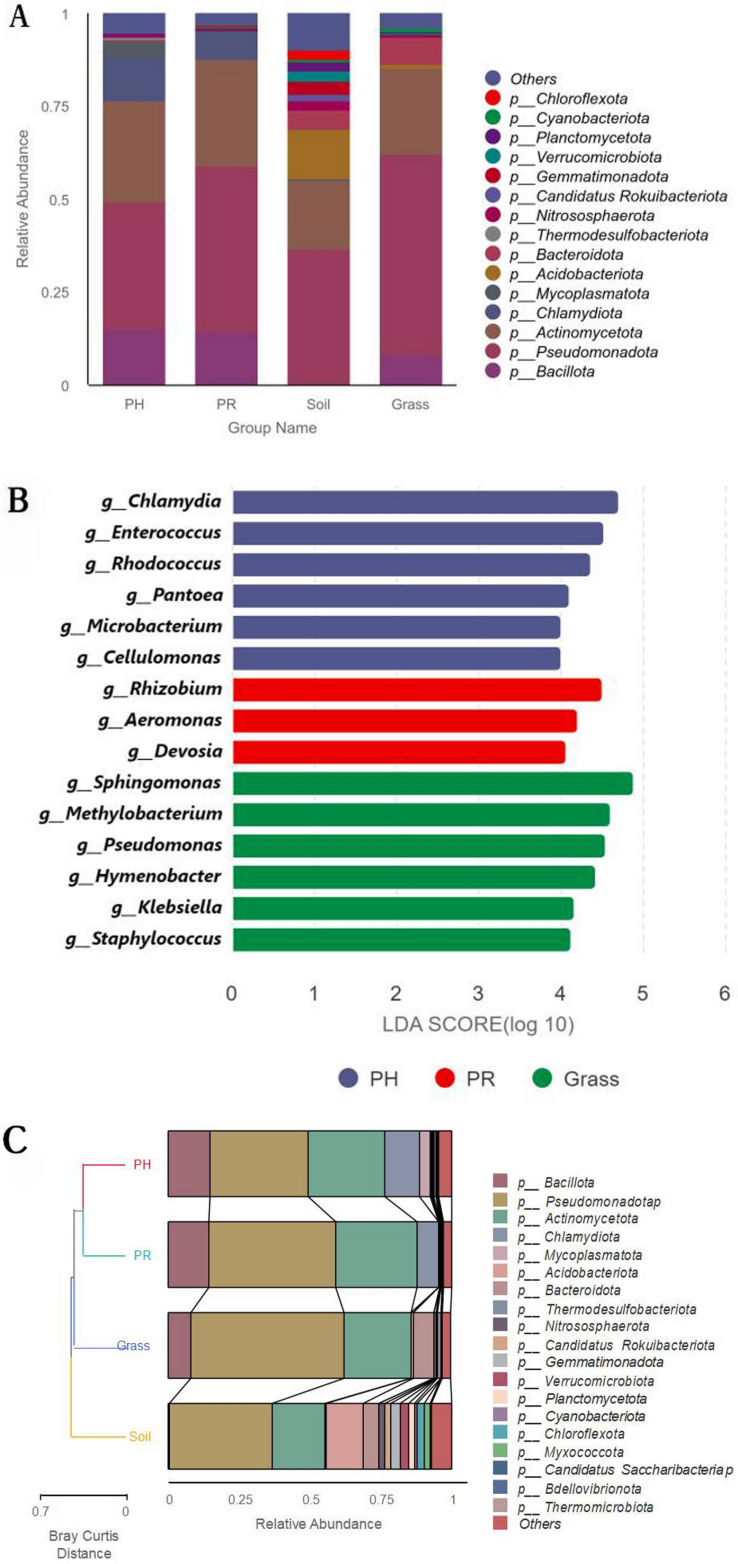
Microbial community structure and phylogenetic disparities across sample groups. **(A)** Relative abundance of bacterial taxa at the phylum level for the PH, PR, grass, and soil groups. Each bar represents one sample group, with colors indicating different phyla. **(B)** Dendrogram illustrating beta-diversity based on Bray–Curtis dissimilarity, accompanied by stacked bar plots showing the corresponding phylum-level composition for each sample. **(C)** Distribution of Linear Discriminant Analysis (LDA) scores for taxa identified as significantly differentially abundant between groups by LEfSe analysis (LDA score > 4.0). The length of each bar represents the effect size of the differential abundance.

Taxonomic annotation of the metagenomic sequencing data against the NR database revealed that Bacteria dominated the microbial community and presented significantly greater phylogenetic diversity than did Archaea, Eukaryota, and viral sequences (Supplementary File 3). A substantial proportion (40–60%) of sequences remained unclassified at the kingdom level. This has been a common scenario in metagenomic studies of non-model organisms and extreme environments, likely attributable to the vast microbial diversity that has not yet been represented in reference databases. The gut microbiota of both PH and PR groups was dominated by the same top four phyla: *Pseudomonadota* (PH: 34.56%; PR: 44.74%), *Actinomycetota* (PH: 26.98%; PR: 28.68%), *Bacillota* (PH: 14.87%; PR: 14.21%), and *Chlamydiota* (PH: 11.98%; PR: 7.73%) (Figure 1A; Supplementary File 4). Unclassified sequences and low-abundance sequences were categorized as “Others,” accounting for 5.08 and 3.26% of the total community in each group, respectively, indicating the presence of a significant number of unknown bacteria in the guts of these two bird species. *Pseudomonadota* and *Actinomycetota* were the dominant phyla in both soil (36.48 and 18.40%, respectively) and grass samples (54.08 and 23.28%, respectively). Subsequent community composition differed: soil was characterized by *Acidobacteriota* (13.23%) and *Bacteroidota* (5.40%), while grass contained *Bacillota* (8.11%) and *Bacteroidota* (7.49%). At the genus level, *Chlamydia* represented the most abundant bacterial taxon in both avian species, with relative abundances of 11.97% (PH) and 7.73% (PR), while host-specific secondary genera emerged—*Enterococcus* (6.60%) and *Rhodococcus* (4.96%) in PH versus *Rhizobium* (5.98%) and *Enterococcus* (5.19%) in PR. *Sphingomonas* was detected at high relative abundances in both soil (6.11%) and grass (14.32%) samples (Supplementary Figure S2; Supplementary File 5). Comparative analysis at the genus level revealed significant disparities in bacterial communities between the PH and PR groups, as detailed in Figure 1B. The LEfSe analysis pinpointed several key discriminative genera that were differentially enriched in each group. Hierarchical clustering analysis revealed that bacterial communities in the feces of Tibetan PH and PR clustered closely between species and were obviously distinct from other environmental communities (Figure 1C). Moreover, the grass and soil samples were grouped together.

**Figure 2:**
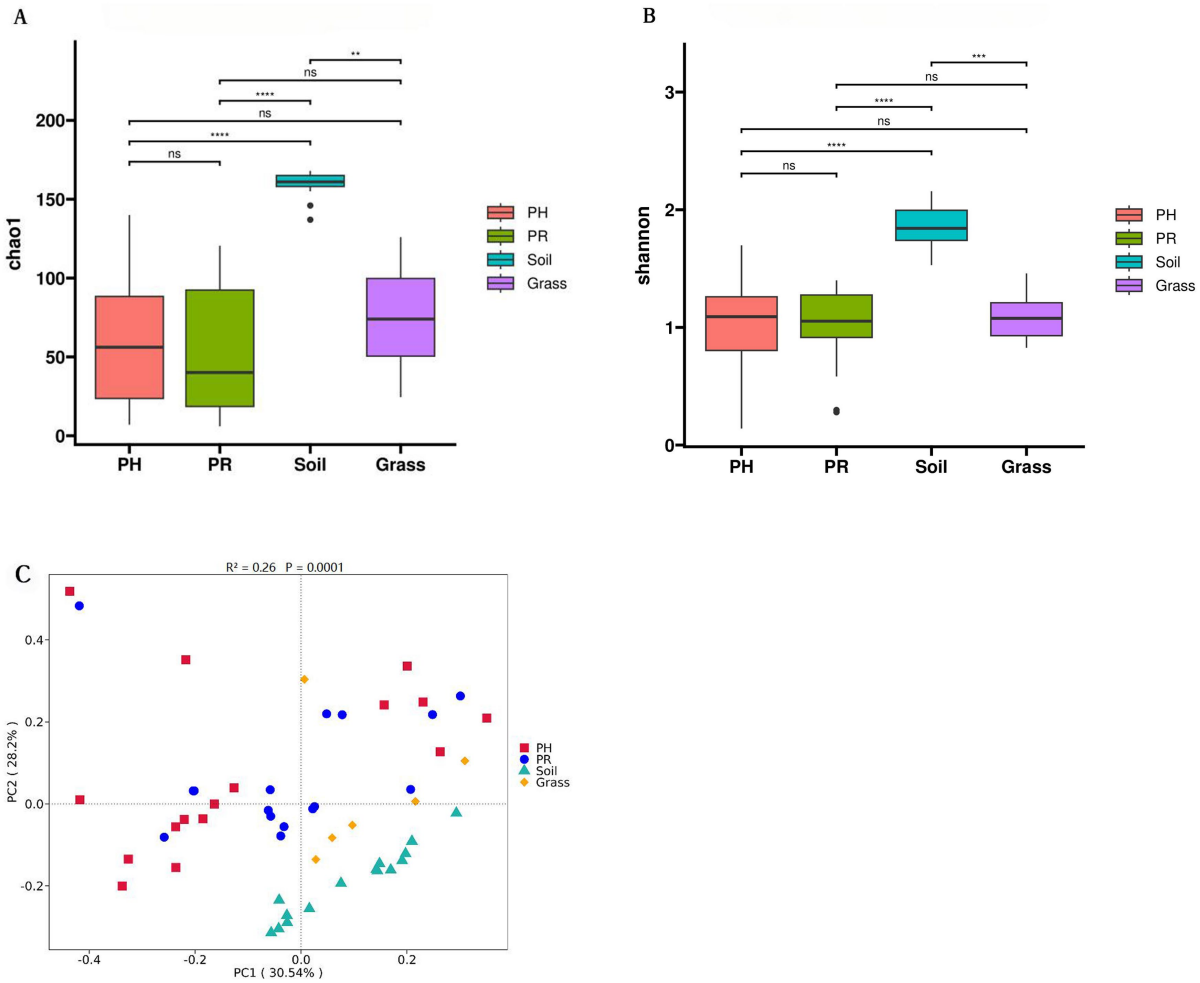
Microbial community structure and phylogenetic disparities across sample groups at the phylum level. **(A)** Chao1 richness and **(B)** the Shannon index, for the PH, PR, grass, and soil groups. **(C)** Principal coordinate analysis (PCoA) based on Bray–Curtis dissimilarity. Boxes denote the interquartile (IQR) between the first and third quartiles (25th and 75th percentiles, respectively) and the line inside denotes the median. Whiskers denote the lowest and highest values within 1.5 times and the IQR from the first and third quartiles, respectively. The asterisks on the top indicate *p < 0.05, **p < 0.01, and ***p < 0.001, ****p < 0.0001; ns, not significant, (Kruskal-Wallis test) (PH: *Pseudopodoces humilis*; PR: *Pyrgilauda ruficollis*).

Venn diagrams revealed a conserved gut microbial architecture between PH and PR (Supplementary Figure S3), despite divergent abundance patterns in specific taxa. The *α*-diversity indices indicated comparable microbial community complexity among wild birds. However, when comparing birds to environmental matrices, both richness and diversity were lower in birds than in soil, while neither showed a significant difference compared to grass (Figures 2A,B). To clarify microbial differences across ecological niches, PCoA of metagenomic data from wild bird feces and adjacent environments, validated by PERMANOVA, revealed the intestinal microbiota of most passerine hosts (PH) exhibited compositional convergence with the predominant microbial profile of proximal environmental reservoirs (PR) at the phylum level (Figure 2C). Significant divergence emerged between specific PH species and PR cohorts, suggesting host phylogenetic constraints on microbial assembly. Subsequent genus-level PCoA corroborated these macroecological patterns while resolving finer taxonomic stratification among niches (Supplementary Figure S4).

In total, 215 opportunistically pathogenic species were identified by comparison against a previously published pathogen list (Li et al., 2015) (Supplementary File 6). *Salmonella enterica*, *Pseudomonas aeruginosa*, and *Escherichia coli* were the most prevalent bacteria. Furthermore, several clinically critical pathogens, such as *Staphylococcus aureus*, *Yersinia pestis*, and *Vibrio cholerae*, were consistently observed.

### 3.2 Functional profiling of the gut metagenome

**Figure 3:**
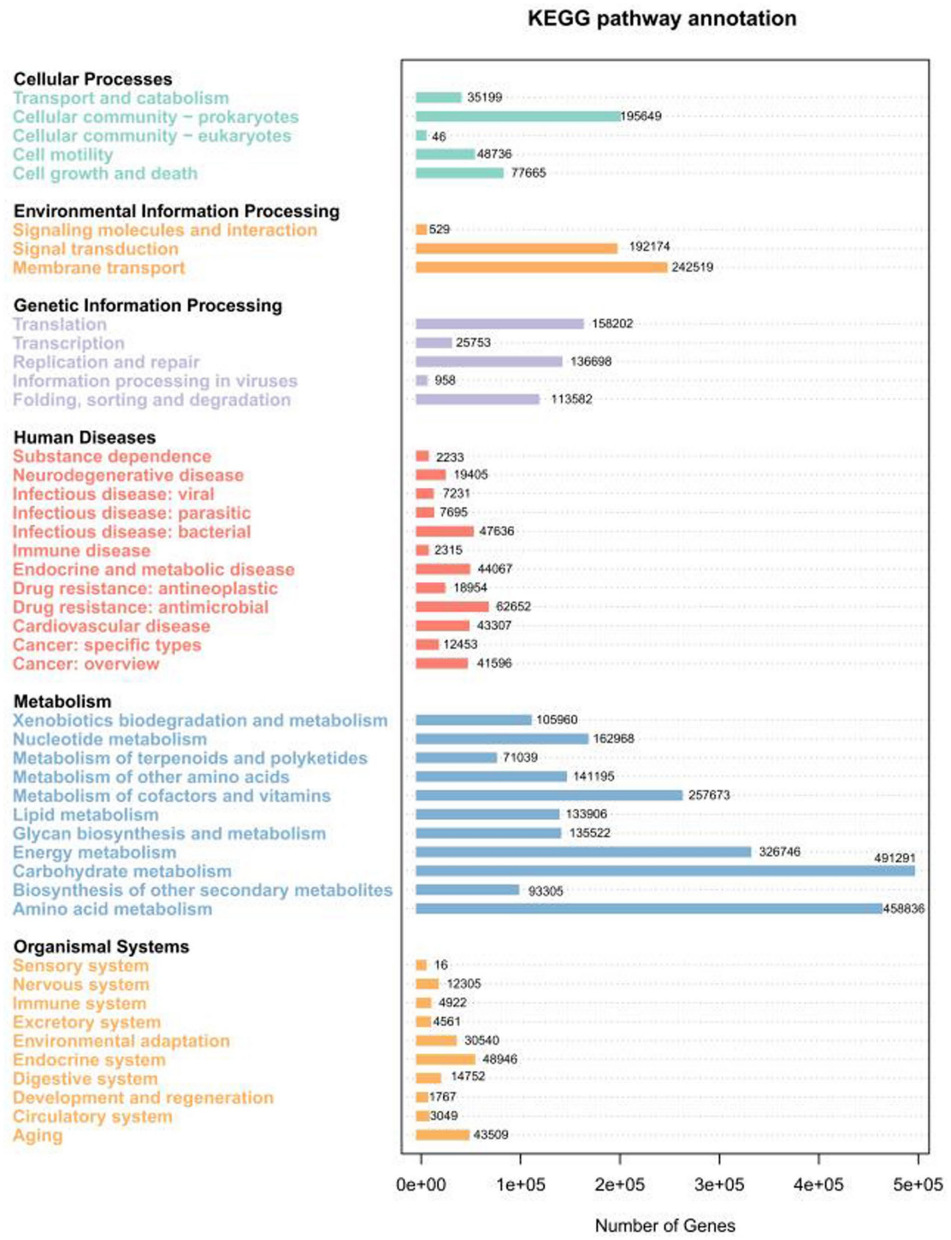
Summary of unigenes matched to KEGG functional categories (level 1 and level 2) present in the metagenomic datasets. The bar plot shows the relative abundance of genes assigned to level 2 KEGG pathways (KEGG: Kyoto Encyclopedia of Genes and Genomes).

**Figure 4:**
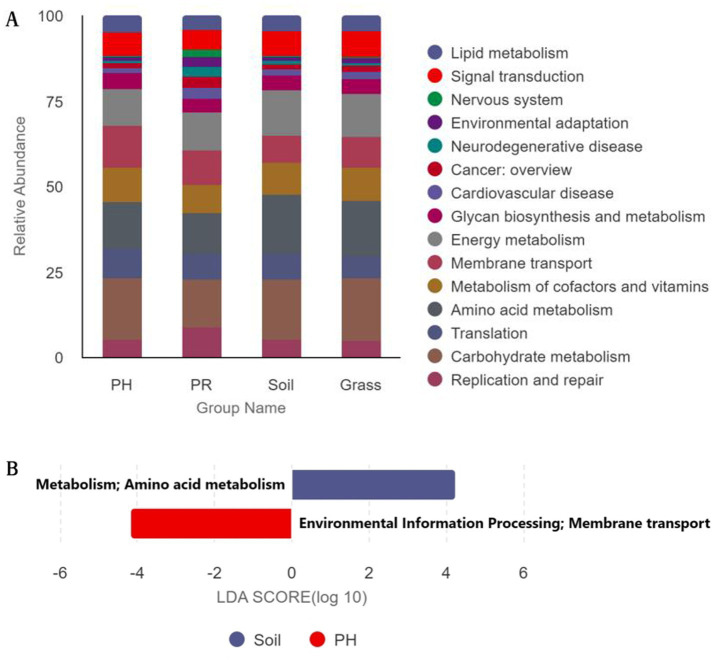
Comparison of KEGG functional profiles. **(A)** Relative abundance of KEGG Orthology (KO) categories at level 2 across the PH, PR, grass, and soil groups. **(B)** Linear discriminant analysis (LDA) effect size scores for KEGG pathways identified as significantly discriminative among groups (LDA score > 4.0). (PH: *Pseudopodoces humilis*; PR: *Pyrgilauda ruficollis*).

The metabolic and functional pathways of the nonredundant (NR) gene catalogue derived from the microbiome were annotated using the KEGG database. Approximately 50% of the NR genes (9,183 KEGG Orthology (KO) functions) were mapped to 410 KEGG pathways level 3 and were then classified into 46 secondary KEGG pathways level 2 (Figure 3). Among the annotated functions, metabolic pathways dominated, accounting for 69.87% of total KO assignments, followed by genetic information processing (23.96%) and environmental information processing (21.08%). Metagenomic KEGG Level 2 functional annotation revealed variations in functional category distribution among the four sample groups (Figure 4A; Supplementary File 7). The top two dominant functional categories were consistent across all groups: Carbohydrate metabolism and Amino acid metabolism. Specifically, Carbohydrate metabolism accounted for 4.62, 4.23, 5.74, and 4.99% in PH, PR, Soil, and Grass, respectively; Amino acid metabolism occupied 3.59, 3.50, 5.66, and 4.43% in the four groups, with the Soil group showing the highest proportions in both categories. Notably, the third dominant category differed between PH and the other three groups: Membrane transport ranked third in PH (3.15%), whereas Energy metabolism was the third in PR (3.30%), Soil (4.31%), and Grass (3.38%). For the fourth dominant category, Metabolism of cofactors and vitamins was predominant in PH (2.58%), Soil (3.07%), and Grass (2.59%), whereas Replication and repair (2.54%) took the fourth place in PR. Among these KOs, the relative abundances of ABC transporters (7.46%) and Two-component system (5.61%) were at the highest level in each sample group (Supplementary File 8). Furthermore, Bray–Curtis distance analysis based on the microbial functional module composition revealed similar clustering patterns among the samples (Supplementary Figure S5).

LEfSe analysis of the compositions and relative abundances of KEGG pathways in the four sample groups gut microflora showed that except for “Amino acid metabolism” and “Membrane transport” (Figure 4B), there were no significant functional differences detected in all the level 2 functional compositions. There were no differences detected in the level 3 functional composition among the four sample groups of gut microbes. The alpha diversity of KEGG functional profiles mirrored the pattern observed for taxonomic (phylum-level) alpha diversity across the sample groups. (Supplementary Figures S6A,B). PCoA revealed distinct functional profiles among the four sample groups. Notably, the PR group exhibited the highest within-group heterogeneity (dispersion), while some individuals from both avian species clustered closely with surrounding environmental samples, indicating functional convergence across these niches (Supplementary Figure S6C).

### 3.3 Antibiotic resistance profiles

**Figure 5:**
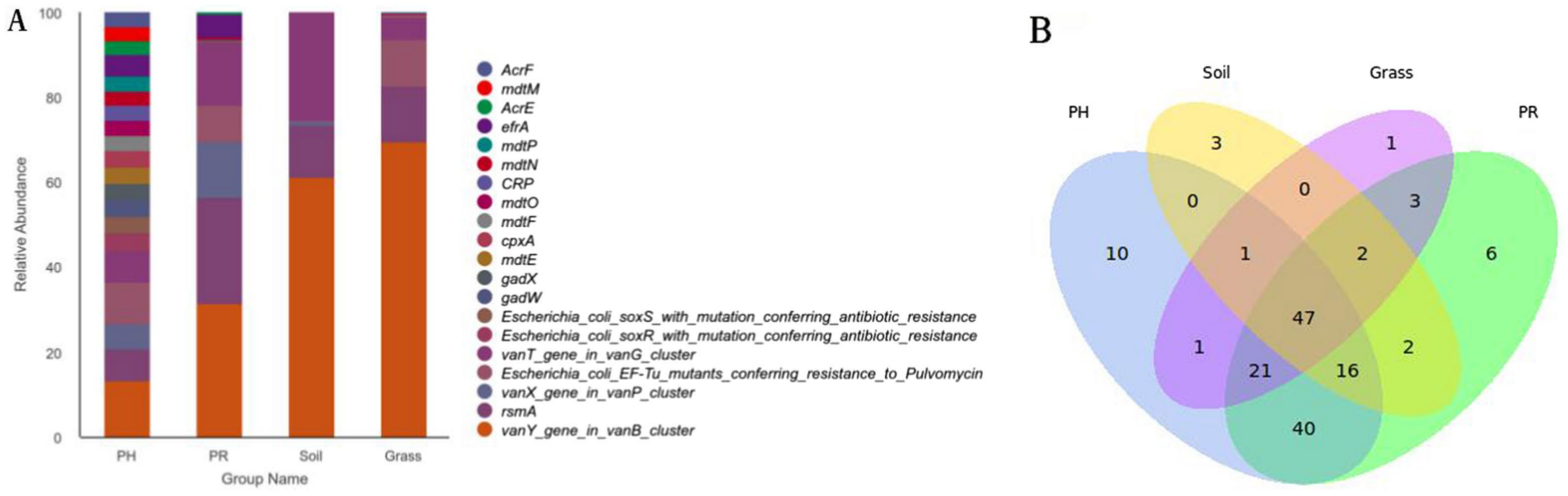
Distribution and composition of ARG in the avian gut and environmental microbiota. **(A)** Relative abundance of the top 20 ARO terms in each sample group. **(B)** Venn diagram showing the shared and unique antibiotic resistance ontologies (AROs) among the PH, PR, grass, and soil samples (PH: *Pseudopodoces humilis*; PR: *Pyrgilauda ruficollis*).

Annotation against the CARD (v3.2.5) identified a total of 153 unique ARO categories across the four metagenome sample groups (Supplementary File 9). The 20 most abundant AROs across samples revealed that the fecal samples from PR presented relatively high ARG abundances, followed by the PH samples (Figure 5A). Most of these genes represented antibiotic target alterations, encompassing four classical glycopeptide resistance gene clusters, namely, the *vanI* cluster, *vanB* cluster, *vanG* cluster, and *vanP* cluster, which confer resistance to glycopeptide and tetracycline antibiotics. The second most common function was antibiotic efflux, represented by three types of classic multidrug efflux pumps: the resistance-nodulation-cell division (RND)-type, ATP-binding cassette (ABC)-type and major facilitator superfamily (MFS)-type, which confer resistance to fluoroquinolones, macrolides, diaminopyrimidine, phenicol, lincosamides, nucleosides, and rifamycin, as well as disinfecting agents and antiseptics. Antibiotic-target alterations were more enriched in the PH resistome, whereas antibiotic efflux mechanisms were more prevalent in the PR resistome.

**Figure 6:**
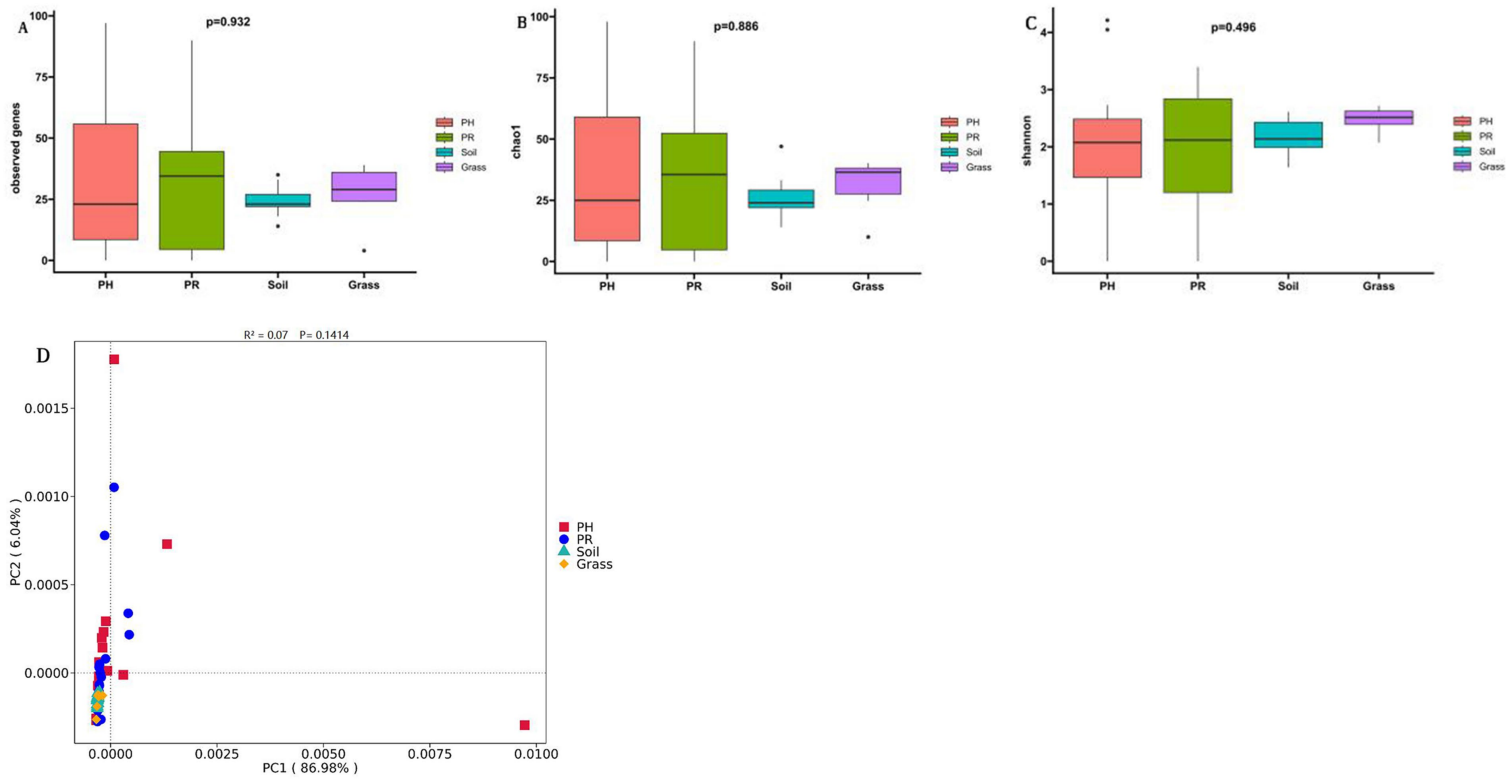
Convergence in alpha and beta diversity of the antibiotic resistome in sympatric plateau birds and its associated environmental microbiota. **(A)** Observed ARG richness **(B)** Chao1 richness index and **(C)** Shannon diversity index of resistance gene composition across sample groups. **(D)** Principal coordinate analysis (PCoA) was performed using Bray–Curtis distances based on CARD annotations. Boxes denote the interquartile (IQR) between the first and third quartiles (25th and 75th percentiles, respectively) and the line inside denotes the median. Whiskers denote the lowest and highest values within 1.5 times and the IQR from the first and third quartiles, respectively (Kruskal-Wallis test) (PH: *Pseudopodoces humilis*; PR: *Pyrgilauda ruficollis;* CARD: Comprehensive Antibiotic Resistance Database).

Alpha diversity analysis revealed convergent richness and diversity of the resistome, with no significant differences observed in the number of ARGs, Chao1, or Shannon indices among the four groups (Figures 6A–C), indicating a homogeneous resistome landscape at the community level. PCoA analysis revealed significant overlap between avian and environmental (soil/grass) resistomes (Figure 6D). Notably, avian samples presented greater *β* diversity than did environmental samples. To investigate the compositional structure underlying this convergent pattern, we further analyzed the sharing profiles of the detected ARGs. The avian-associated samples PR and PH dominated the resistome, containing 137 and 136 AROs, respectively, and collectively representing approximately 90% of the total, among which 47 AROs were shared with the environmental samples (Figure 5B). Notably, 124 AROs (90% of avian-associated AROs) were common to both the PH and PR, indicating conserved resistance gene pools between these sympatric passerines.

**Figure 7:**
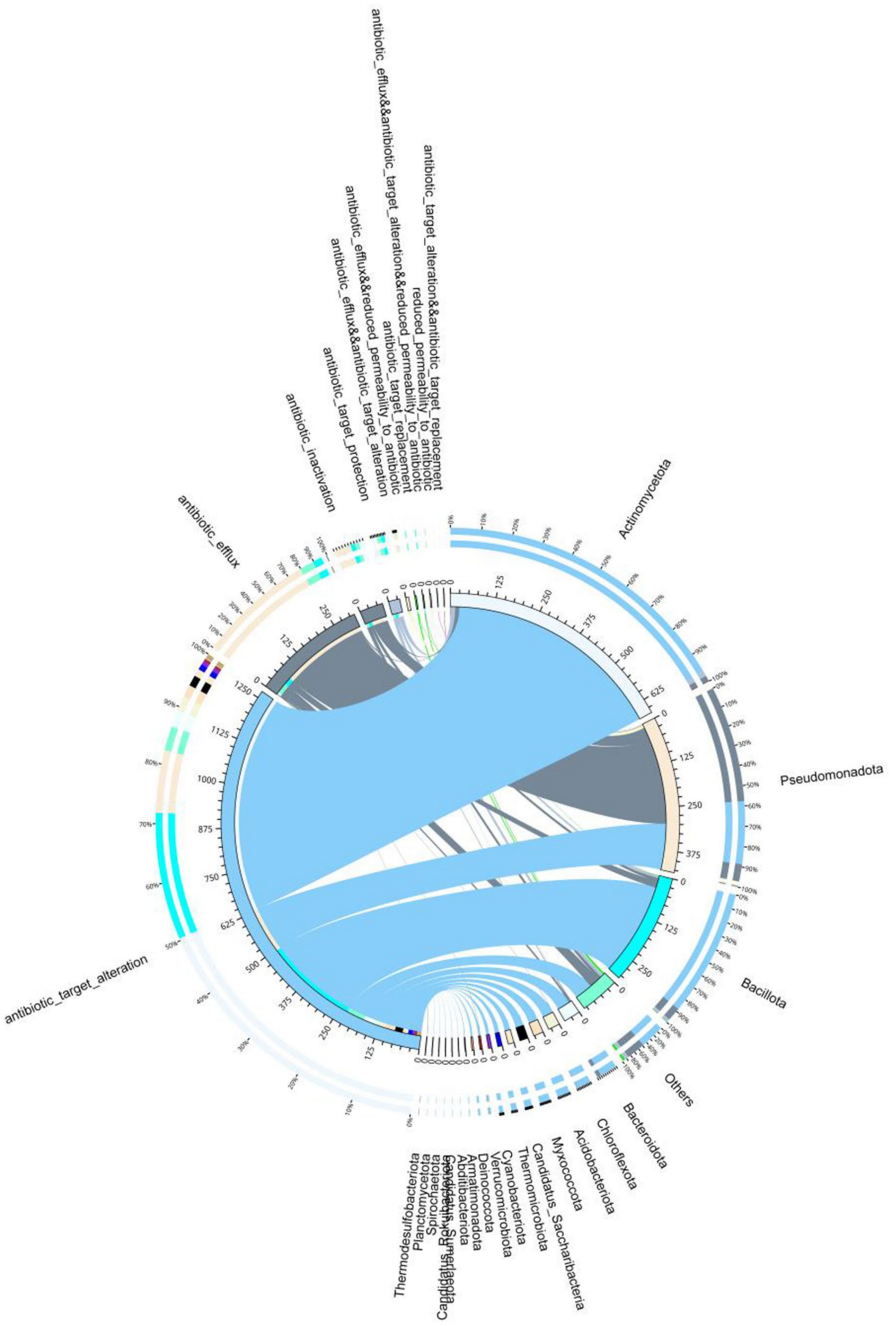
Circos plot illustrating the associations between bacterial taxa and antibiotic resistance mechanisms in the metagenomic dataset.

To systematically elucidate the associations between bacterial communities and antibiotic resistance mechanisms within the samples, we generated a double-circle plot depicting the relationships between metagenomic bacteria and resistance mechanisms (Figure 7). Antibiotic target alteration has emerged as the most prevalent resistance mechanism, exhibiting extensive associations with multiple bacterial phyla and demonstrating significant enrichment within *Pseudomonadota*. Furthermore, antibiotic efflux was also highly prevalent in both *Pseudomonadota* and *Bacillota*, indicating that efflux pumps represent a common resistance strategy in these taxa. Additionally, certain phyla, including *Bacteroidota, Chloroflexota,* and *Verrucomicrobiota*, exhibited moderate associations with multiple resistance mechanisms. This pattern reflects the diverse adaptation strategies employed by bacterial populations in response to antibiotic pressure in the sampled environment.

**Figure 8:**
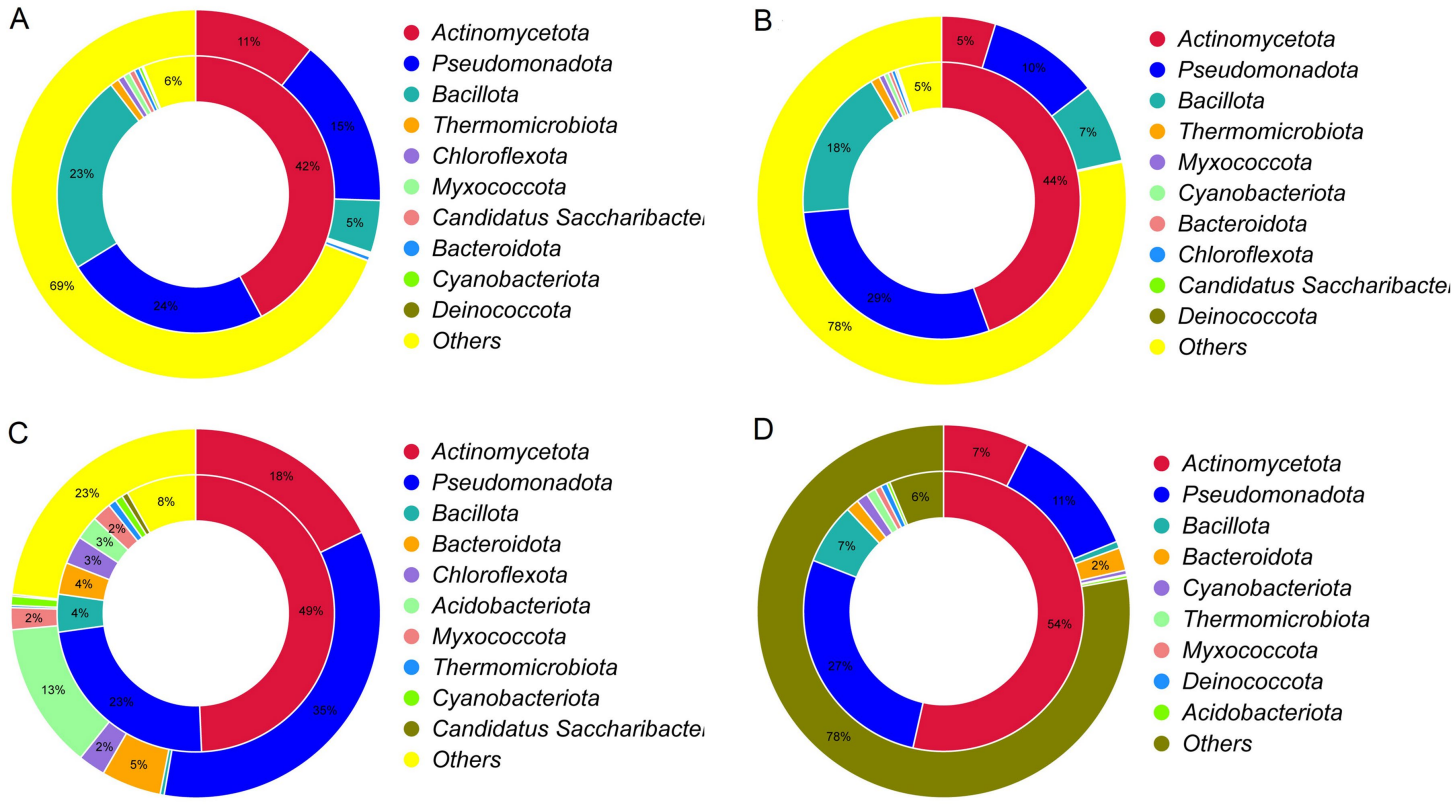
**(A–D)** Circos plots representing the alignment of the proportions of different antibiotic resistance ontologies and microbial phyla in the PH **(A)**, PR **(B)**, soil **(C)**, and grass **(D)** groups. The inner ring shows the distribution of different antibiotic resistance ontologies in the corresponding microbial phyla. The outer ring shows the relative abundance of different phyla in each group (PH: *Pseudopodoces humilis*; PR: *Pyrgilauda ruficollis*).

These AROs were further analyzed for their microbial origin. Taxonomic analysis revealed that the AROs were predominantly associated with *Actinomycetota*, *Pseudomonadota* and *Bacillota* across all the samples (Figures 8A–D). Specifically, in the PH group, *Actinomycetota* (11% relative abundance), *Pseudomonadota* (15%), and *Bacillota* (5%) contributed 42, 24, and 23% of ARGs, respectively. In the PR group, these three phyla (5, 10, 7% abundance) contributed 44, 29, and 18%. In the Soil group, they (18, 35, 0.35% abundance) contributed 49, 23, and 4%. In the Grass group, they (7, 11, 0.59% abundance) contributed 54, 27, and 7%. Additionally, *Acidobacteriota*, which was more abundant (13%) in the soil samples, contributed only 2.8% of the total ARGs. These findings underscore a systemic disproportionality between microbial prevalence and resistance gene contribution, where phylogenetically restricted taxa drive ARG reservoirs despite their low ecological representation.

## 4 Discussion

This study provides the first systematic characterization of gut microbiome adaptation and ARG dissemination in two plateau-endemic passerines—*Pseudopodoces humilis* (PH) and *Pyrgilauda ruficollis* (PR)—and their surrounding environmental samples under extreme conditions on the Qinghai–Tibet Plateau. These species were selected not only for their ecological representativeness as endemic taxa but also for their dual importance in elucidating extremophilic adaptation and ARG transmission dynamics. Taxonomic analysis revealed no significant divergence in the gut microbial community structure at the phylum level between species, with *Actinomycetota*, *Bacillota* and *Pseudomonadota* dominating both assemblages, which is consistent with the typical passerine microbiota (Lewis et al., 2016; Kropáčková et al., 2017; Joakim et al., 2023). This similarity likely arises from their shared ecological niche as cave-dwelling species and partial dietary overlap, underscoring the roles of host ecology, diet, and environmental selection in shaping microbial communities, in accordance with the host–microbe coevolution theory (Moeller et al., 2016; Maritan et al., 2024). High-altitude environments appear to drive the enrichment of specific microbial taxa, which may be closely linked to the host’s adaptive strategies. For example, a study on two high-altitude breeding populations (*Charadrius alexandrinus* and *Charadrius atrifrons*) on the Qinghai-Tibet Plateau showed that despite interspecific differences, their gut microbiota converged to a community structure dominated by *Bacillota* and *Pseudomonadota* (Sun et al., 2024). This finding has also been observed in previous studies on high-altitude animals in the Qinghai-Tibet Plateau, including yaks, Tibetan antelopes, pikas, and Tibetan chickens (Li et al., 2016; Ma et al., 2019; Bhagat et al., 2025; Yan et al., 2025). In contrast, in low-altitude environments, avian gut microbiota is typically characterized by a higher relative abundance of Bacteroidota than Firmicutes (Bo et al., 2025; Sun et al., 2024). A higher Firmicutes-to-Bacteroidetes ratio in the gut microbiota is associated with enhanced energy harvest from diet, thereby aiding animals in maintaining metabolic balance and body homeostasis under high-altitude conditions (Bhagat et al., 2025; Sun et al., 2024). Both richness and diversity were lower in birds than in environmental samples. Previous studies have shown that as altitude increases, the diversity and richness of the intestinal microbiome in birds decrease (Joakim et al., 2023), which to some extent led to the reduction in the diversity and richness of the intestinal microbiome in the bird groups. Members of the phylum *Bacillota* (e.g., Butyrivibrio spp.) mediate the degradation of insoluble fibers (e.g., cellulose and resistant starch) and produce butyrate, conferring anti-inflammatory benefits and enhancing intestinal barrier integrity. These metabolic functions enable hosts to extract energy and nutrients from carbohydrates, polysaccharides, sugars, and fatty acids (Flint et al., 2008; Tap et al., 2009; Berry, 2016). At the genus level, *Chlamydia* was predominant in both birds. As obligate intracellular bacteria, *Chlamydia* species employ a type III secretion system (T3SS) to translocate effector proteins for hijacking host cell functions and evading immune clearance, a strategy that may facilitate their persistence in wild bird populations, while they are also established pathogens causing human diseases such as pulmonary infections, trachoma, vaginitis, intestinal inflammation, diarrhea, and abdominal pain (Elwell et al., 2016; Wang et al., 2023). The *Enterococcus* genus frequently exhibits multidrug resistance (particularly to *β*-lactams and quinolones), highlighting its ARG dissemination potential (Fujii et al., 2024). Key distinctions included elevated abundances of *Rhodococcus* (4.96%) and *Nocardioides* (4.11%) in PH, which aligned with their relatively high proportions of *Pseudomonadota* (26.98%). Conversely, PR presented relatively high abundances of plant/soil-associated genera (e.g., *Rhizobium*: 5.98%; *Rhodococcus*: 4.29%). This suggests greater grass seed consumption in PR, with their microbiota more substantially influenced by environmental bacteria (e.g., soil- and plant-derived microbes) (Mikaelyan et al., 2015). In support of this, the soil samples presented high abundance of *Acidobacteriota* (13.23%), a diverse and ubiquitous soil phylum linked to local geochemical properties (Barns et al., 1999; Cole et al., 2005). The microbial structure of birds is different from that of soil, and the phylogenetic composition of the soil microbiota is less heterogeneous than that of the bird microbiota and grass microbiota, potentially because of the diverse and variable conditions in the environmental samples. The diversity and composition of soil bacterial communities, on a large spatial scale, can be predicted largely by a single variable: soil pH (Fierer and Jackson, 2006).

The overall functional profiles revealed that the microbial gene pool in both avian gut and environmental samples was dominated by metabolic pathways, which accounted for 69.87% of total KO assignments. Among these, carbohydrate metabolism, amino acid metabolism, energy metabolism, and metabolism of cofactors and vitamins were the most dominant functional categories. These results were consistent with previous findings that the gut microbiota of *Passer ammodendri* in high-altitude (Pamir Plateau) exhibited significantly enhanced carbohydrate and energy metabolic functions compared to those at low altitudes, facilitating hosts to maximize energy extraction from limited food resources (Liu et al., 2025). During the vertical migration of the *Tarsiger rufilatus*, its gut microbiota was initially enriched in pathways for carbohydrate and cofactor/vitamin metabolism in autumn, and subsequently exhibited enhanced amino acid metabolism in response to colder temperatures and resource scarcity, collectively improving energy extraction and nutrient utilization efficiency (Zhang et al., 2024). Our results strongly confirm that the functions of microbial communities and their interactions with hosts and the environment collectively promote their ability to cope with environmental changes. In addition, the “Membrane transport” function was highly enriched in PH and PR, while “Replication and repair” in PR was significantly more abundant than that in PH. ATP-binding cassette (ABC) transporters, the most strongly expressed pathway in membrane transport, are directly involved in ATP generation. Low oxygen levels and high ultraviolet radiation may cause DNA and protein impairment in high-altitude environments, and genes related to replication and repair are conducive to reduce the damage of biological molecules (Wu et al., 2020). Such functional differences may stem from subtle variations in microhabitat selection, specific diet, or behavioral patterns between the two bird species, ultimately shaping distinct microbial functional characteristics through host selection. However, a notable difference is that environmental samples (especially soil) had significantly higher relative abundances of these core metabolic pathways than avian gut samples in this study. This strongly suggests that in the extreme, nutrient-scarce Qinghai-Tibet Plateau environment, the environmental microbial pool undertakes more active ecological functions of organic matter decomposition and primary nutrient transformation, thereby providing a basic energy and material cycling platform for the entire ecosystem (including herbivorous/omnivorous birds).

We further elucidated the microbial risks associated with PH and PR and their surrounding environmental samples, in which 215 opportunistic pathogens were detected. The identification of such a high abundance of opportunistic pathogens underscores the complexity of their microbial communities’ pathogenic potential and suggests that wild birds may serve as potential reservoirs of infection, highlighting the need for continuous surveillance of these pathogens. Systematic screening of pathogen profiles using metagenomic technology provides novel strategies for disease prevention, control, and early diagnosis while also establishing a more robust data foundation for related pathological studies.

The 21st-century public health challenge posed by the global spread of antibiotic resistance (AR) involves complex environmental drivers in avian species inhabiting the Qinghai–Tibet Plateau. Environmental compartments in China demonstrate pervasive antibiotic contamination, with detection rates reaching 100% in soils, 98.0% in surface waters, and 96.4% in coastal waters (Lyu et al., 2020), and the transmission of ARB through fecal deposition and contaminated water sources (Phan et al., 2013; Ramírez-Martínez et al., 2018; Wille et al., 2021). This study employed metagenomic sequencing to conduct unbiased resistome profiling of PH and PR, circumventing the limitations inherent in targeted detection approaches (Li et al., 2020). We comprehensively identified 153 ARO categories. While both species shared >90% of the ARGs, PR had a significantly greater total ARG abundance. Conceivably, depending on the host bacterium, ARGs could possess other functions not directly related to antibiotic resistance, which could contribute to their abundance in different environments (Martínez, 2008; Dantas and Sommer, 2012). The gut microbiomes of these species have demonstrated resistance to nearly all major classes of antibiotics relevant to clinical and agricultural practice, with glycopeptide-, fluoroquinolone-, and tetracycline-resistance genes constituting the core resistome. Glycopeptide antibiotics, notably vancomycin and teicoplanin, have served as last-resort therapeutics against bacterial infections for more than half a century and remain essential for treating methicillin-resistant *Staphylococcus aureus* (MRSA) and penicillin-resistant *Streptococcus pneumoniae*. The historical use of avoparcin as a livestock growth promoter (Phillips, 2007) likely contributed to the evolution of vancomycin-resistant enterococci (VREs) (Manson et al., 2003; Phillips et al., 2004). Critically, homologous resistance genes (*vanA, B, C, D, E, and G*) have been detected in >10,000-year-old permafrost samples (D'Costa et al., 2011; Wright and Poinar, 2012) and are highly abundant in contemporary soil, marine, and human fecal matrices (Nesme et al., 2014), confirming that environmental reservoirs are intrinsic sources of ARGs. The persistence of fluoroquinolone resistance is linked to environmental stability and low biodegradability (Bhatt and Chatterjee, 2022), whereas tetracycline resistance genes are widely distributed among plateau nonmigratory birds (e.g., corvids and *Gyps himalayensis*) (Zhai et al., 2024; Wang et al., 2025), collectively indicating a regional ecological network of resistance. Our research revealed that the antibiotic resistomes of both high-altitude bird species and their habitats exhibited convergent and homogeneous richness and diversity, despite their distinct ecological niches. This functional convergence, also observed in metabolic pathways, underscores the extreme high-altitude environment as a predominant selective force shaping microbial traits, including resistance. Significant resistome overlap between avian and environmental samples (PCoA), along with higher *β*-diversity in bird guts, indicates frequent bidirectional exchange of resistance genes. Thus, the avian gut likely functions as a dynamic “sink” and “source,” accumulating and potentially disseminating environmental resistance genes. Given the absence of direct antibiotic exposure in both bird species, their ARGs likely originate from environmental pathways [e.g., antibiotic residues from free-grazing livestock enter ecosystems via excreta (Zubair et al., 2023)], with 47 AROs shared between birds and environmental samples (soil, grass), highlighting the critical role of environmental transmission. Substantial quantities of antibiotics discharged from hospitals and livestock operations contaminate rivers, sediments, and soils (Zhang et al., 2015), enabling the transmission of ARB from contaminated matrices to avian species (Bonnedahl and Järhult, 2014). ARGs may be disseminated to hosts via contaminated water, food chains, or aerosols, posing elevated risks in regions with limited public health infrastructure (Munk et al., 2018). This study reveals a core characteristic of the high-altitude ecosystem resistome: ARGs are not uniformly distributed but are highly concentrated within a select few key bacterial phyla, establishing a distinct “low-abundance–high-resistance” paradigm. Specifically, while the combined relative abundance of *Actinomycetota*, *Pseudomonadota*, and *Bacillota* was 31, 22, 53, and 18% in the PH, PR, Soil, and Grass sample groups, respectively, they contributed 89, 91, 76, and 88% of the identified ARGs. A particularly striking example was observed in soil, where *Bacillota*, representing only 0.35% of the community, accounted for 4% of the ARGs—an enrichment efficiency exceeding 11-fold relative to its abundance. This profound disproportionality indicates that the reservoir function of the environment for ARGs is primarily executed by specific taxonomic groups (Pal et al., 2016). The specificity of this paradigm is further underscored by a critical counter-example. In grass samples, *Acidobacteriota*, despite being a dominant phylum (13% abundance), contributed a mere 2.8% of the ARGs, exhibiting a “high-abundance–low-resistance” profile. This sharp contrast confirms that high taxonomic abundance does not predispose a taxon to be a major ARG carrier. Instead, the “low-abundance–high-resistance” trait appears to be a conserved, phylogenetically linked characteristic of *Actinomycetota*, *Pseudomonadota*, and *Bacillota* within this ecosystem. We posit that the aggregation of ARGs in taxa like *Pseudomonadota* is closely tied to their high genomic plasticity and active horizontal gene transfer (HGT) capabilities, positioning them as potential hubs for resistance dissemination (Larsson and Flach, 2022). To transition from observing this paradigm to assessing its risk, future research must transcend correlative descriptions. By employing culturomics coupled with pangenome analysis (Raymond et al., 2019), future studies should focus on deciphering the genomic context of ARGs within these key phyla to distinguish whether they reside in the stable “core genome” or the mobile “accessory genome.” This will directly quantify the potential mobilizable resistance pool, thereby elevating the phenotypic understanding of “low-abundance–high-resistance” phenomenon to a mechanistic comprehension of its inherent “high-transmission-risk” potential.

The present study has several limitations that should be acknowledged. First, the relatively small sample size (*n* = 53) and cross-sectional design constrained our ability to investigate gut microbiota dynamics and their impacts across the host life cycle. Future studies should incorporate broader taxonomic representations, encompassing both migratory and resident avian populations across diverse ecosystems, to systematically elucidate the ecological and evolutionary drivers shaping microbial communities and their role in antibiotic resistance transmission. Longitudinal monitoring is particularly essential to resolve the effects of seasonal fluctuations, environmental stressors (e.g., extreme climatic events, variations in food resources), and migratory behavior on microbial community structure and resistome dynamics. Additionally, annotations relying on the NR and CARD databases are inherently subject to taxonomic bias toward well-characterized taxa, potentially overlooking plateau-specific ARGs or novel resistance mechanisms. Therefore, integrating culturomics with single-cell sequencing technologies represents a critical approach for characterizing the biological traits and resistance potential of uncultivated/unclassified microorganisms, thereby advancing the current understanding. Furthermore, the absence of quantitative data on environmental factors (e.g., antibiotic residue concentrations, pH, and heavy metal pollution) impedes the precise delineation of the environmental drivers underpinning the resistome. Future investigations should employ multivariate statistical modeling frameworks (e.g., structural equation modeling and machine learning algorithms) to quantify causal relationships between multiple environmental stressors and the evolution of microbial antibiotic resistance.

## 5 Conclusion

On the basis of metagenomic analysis of nonmigratory passerines and their habitats on the Qinghai Tibetan Plateau, this study revealed significant convergence in gut microbial composition and function across species. These birds represent critical reservoirs of ARGs, which serve as major sources of resistance determinants for local environmental bacteria. Transmission pathways were clearly demonstrated by the sharing of 47 resistance determinants between avian hosts and proximal environmental matrices (soil/grass), combined with livestock-derived antibiotic influx via excreta. These mechanisms establish the Tibetan Plateau as a hotspot for resistance gene dissemination. Collectively, these findings reposition resident passerines as sentinel species for ecosystem health and bridge the need for cross-boundary AMR transmission. Mitigating global AMR thus necessitates interdisciplinary strategies targeting environmental reservoirs (e.g., regulating antibiotic use in livestock operations) and monitoring avian-mediated gene flow.

## Data Availability

The data presented in the study are deposited in the NCBI repository, accession number PRJNA1412623.

## Supplementary material

SUPPLEMENTARY FILE 1 Statistical results of raw sequencing data preprocessing.

SUPPLEMENTARY FILE 2 Summary of assembled scaftigs (≥500 bp) across samples.

SUPPLEMENTARY FILE 3 Relative abundance of taxonomic annotation at the kingdom level across samples.

SUPPLEMENTARY FILE 4 Relative abundance of taxonomic annotation at the phylum level across samples.

SUPPLEMENTARY FILE 5 Relative abundance of taxonomic annotation at the genus level across samples.

SUPPLEMENTARY File 6 Inventory of opportunistic pathogens detected across sample groups.

SUPPLEMENTARY FILE 7 Relative abundance of functional annotation at the KEGG level 2 across samples.

SUPPLEMENTARY FILE 8 Relative abundance of functional annotation at the KEGG level 3 across samples.

SUPPLEMENTARY FILE 9 Antibiotic resistance ontology (ARO) annotation results across samples.
