# Supplementary material for: Convergent gut microbiome adaptation and pervasive antibiotic resistome in Qinghai–Tibet Plateau passerines
Source: Front Microbiol. 2026 Feb 4;16:1733974. doi: 10.3389/fmicb.2025.1733974 (PMC12913512; doi:10.3389/fmicb.2025.1733974)
Supplement: Supplementary file 1 [file Data_Sheet_1.zip › 1733974-Supplementary Material/Supplementary Figure.docx]

**Table S1 The detailed information for the sampling efforts in Qinghai‒Tibet Plateau**

| **Site No.** | **Date** | **Sample type** | **Sample ID** | **Altitude(m)** | **Latitude** | **Longitude** | **Bird types** |
| --- | --- | --- | --- | --- | --- | --- | --- |
| 1 | Jly. 2024 | Bird feces  Soil  Grass | P.h1~5  soil1~5 | 3098.46 | N 37^o^ 37’ 45.53” | E 98^o^ 82’ 39.03” | *Pseudopodoces humilis* |
| 2 | Jly. 2024 | Bird feces  Soil  Grass | P.h6~11  P.r1~7  soil6~10  grass1~3 | 3491.69 | N 37^o^ 63’ 29.58” | E 101^o^ 23’ 24.47” | *Pseudopodoces humilis*  *Pyrgilauda ruficollis* |
| 3 | Aug. 2024 | Bird feces  Soil  Grass | P.h12~16  P.r8~16  soil11~15  grass4~6 | 3760.60 | N 35^o^ 52’ 16.58” | E 99^o^ 41’ 50.20” | *Pseudopodoces humilis*  *Pyrgilauda ruficollis* |


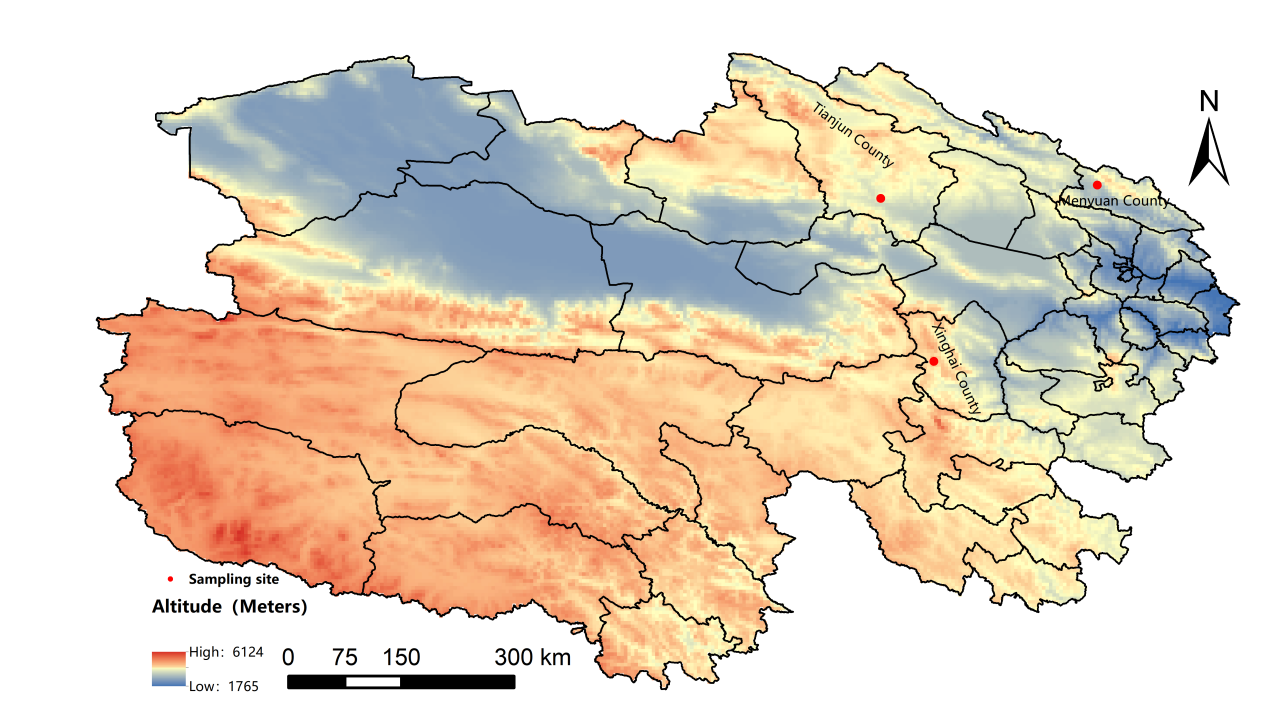


**Figure S1. Illustration of the sampling sites in Qinghai‒Tibet Plateau.**The three sampling locations are indicated by numbered red markers. The map was generated using ArcGIS software (v10.8; Esri, USA) based on cartographic data from the National Fundamental Geographic Information System of China.


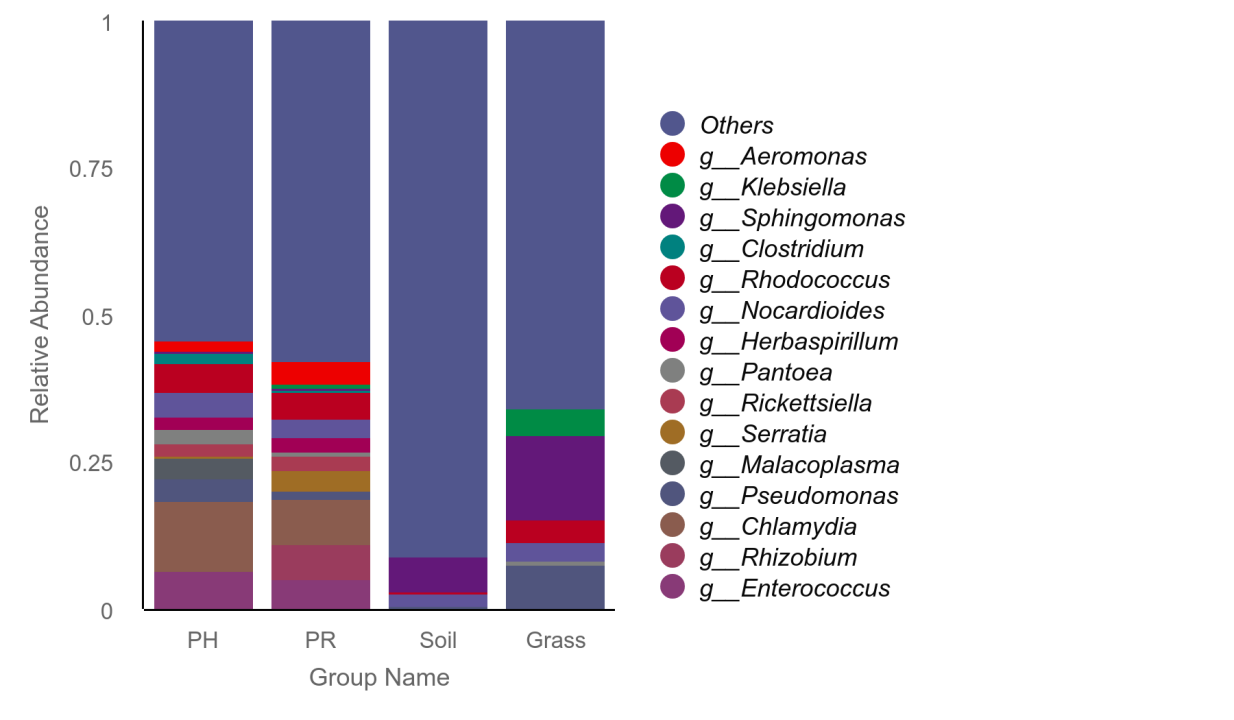


**Figure S2. Taxonomic profiles of the microbial communities at the genus level in each group:Each bar represents one group and is colour-coded by genus**(PH: *Pseudopodoces humilis*; PR: *Pyrgilauda ruficollis*; g: genus).


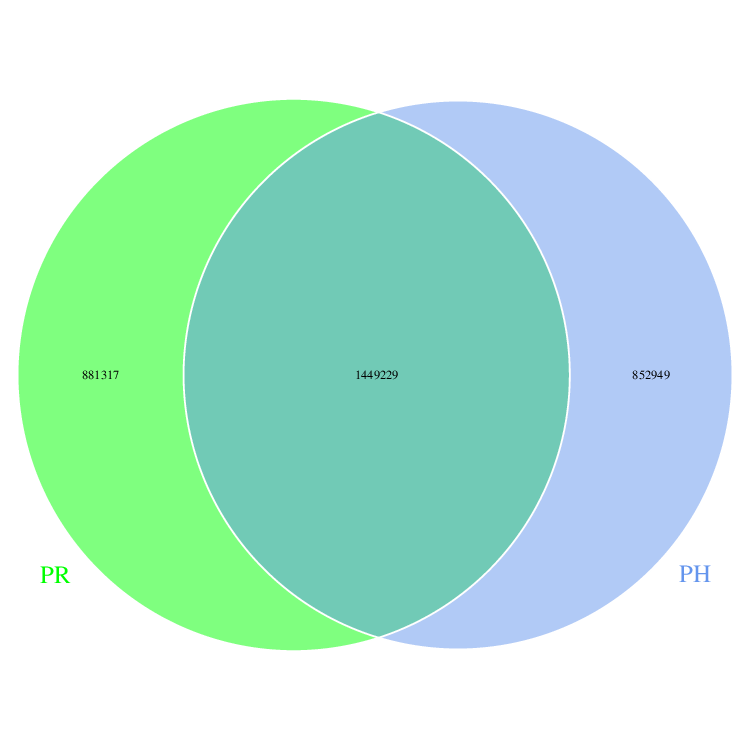


**Figure S3. Venn diagrams showing the unique and shared microbial genera between Group PH and Group PR**(PH: *Pseudopodoces humilis*; PR: *Pyrgilauda ruficollis*).


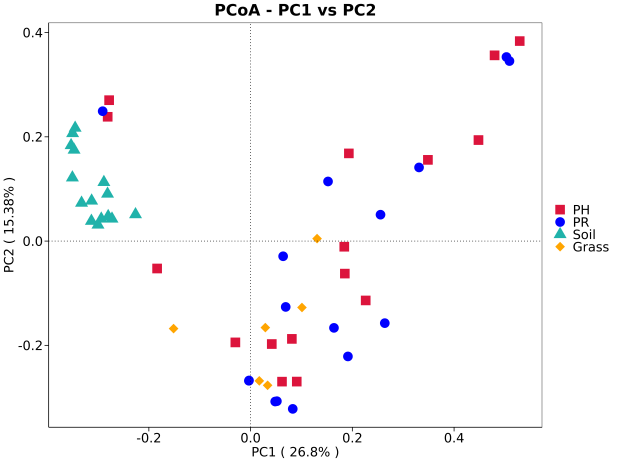


**Figure S4. Dissimilarity of microbial communities among PH, PR, grass and soil samples: Principal coordinate analysis (PCoA) based on genus-level abundance (Bray–Curtis dissimilarity)**(PH: *Pseudopodoces humilis*; PR: *Pyrgilauda ruficollis*).
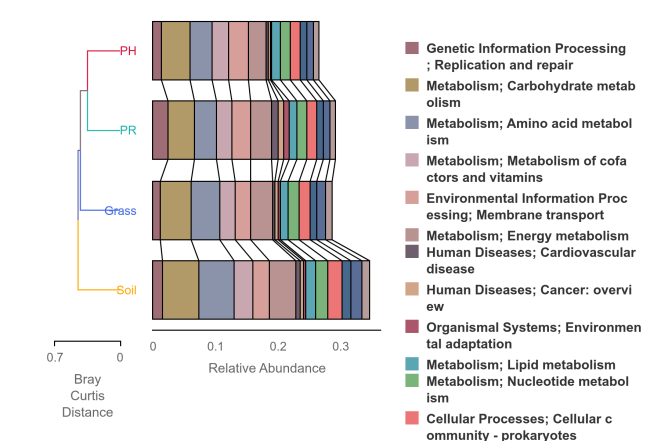


**Figure S5. Hierarchical clustering of KEGG functional profiles and pathway abundance.** Analysis based on Bray–Curtis distance of functional module abundance, with corresponding composition of level 1 and level 2 KEGG pathways shown in stacked bar plots.(PH: *Pseudopodoces humilis*; PR: *Pyrgilauda ruficollis*).


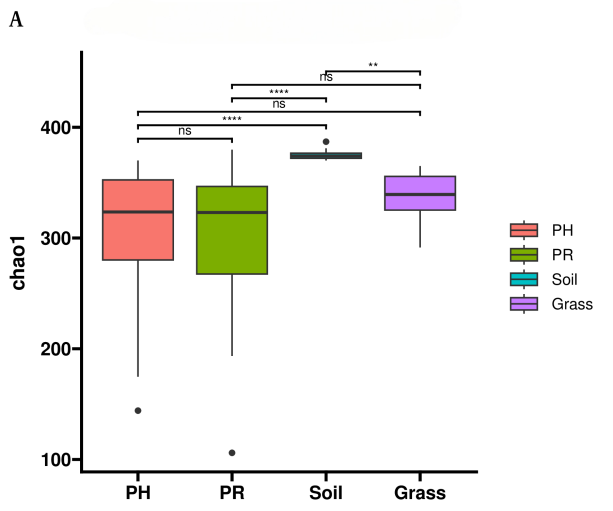

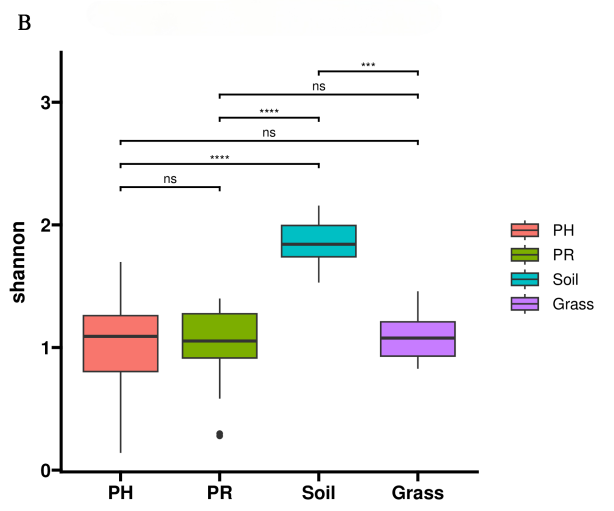


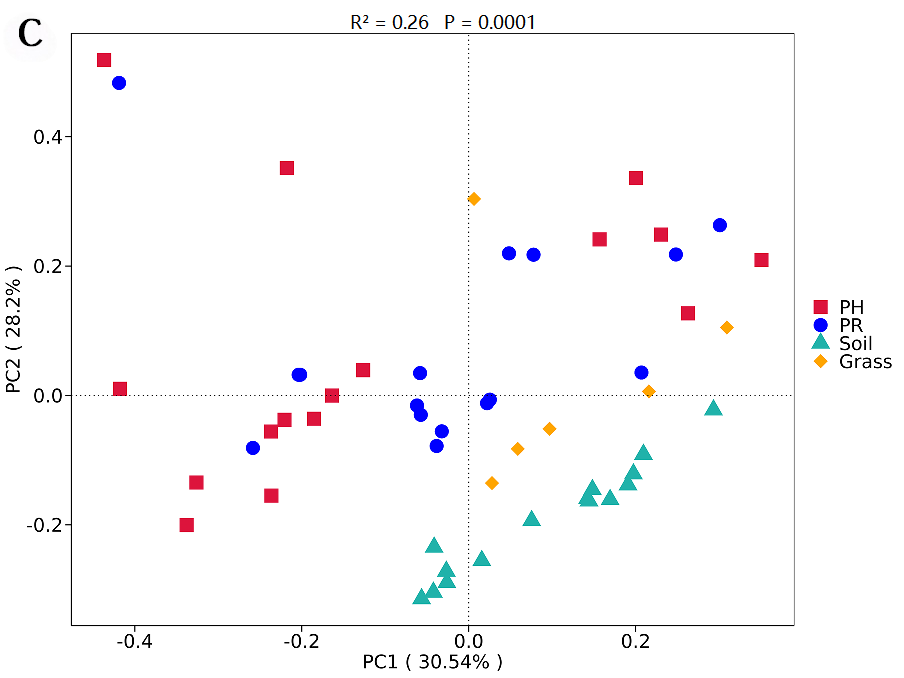


**Figure S6. Diversity of KEGG pathways (Level 3) across sample groups.**(A) Chao1 richness estimator and (B) Shannon index of KEGG Orthology (KO) functional profiles for the PH, PR,Soil and Grass groups. (C) Principal coordinates analysis (PCoA) plot based on Bray–Curtis dissimilarity, showing beta diversity of KEGG functional profiles among groups.Boxes denote the interquartile (IQR) between the first and third quartiles (25th and 75th percentiles, respectively) and the line inside denotes the median. Whiskers denote the lowest and highest values within 1.5 times and the IQR from the first and third quartiles, respectively.The asterisks on the top indicate *P < 0.05, **P < 0.01, and ***P < 0.001, **** p < 0.0001; ns, not significant,(Kruskal-Wallis test) (PH: *Pseudopodoces humilis*; PR: *Pyrgilauda ruficollis*).
